# HOW TO GET AN NIA GRANT

**DOI:** 10.1093/geroni/igad104.0973

**Published:** 2023-12-21

**Authors:** Kenneth Santora

**Affiliations:** NIA, NIH, Bethesda, Maryland, United States

## Abstract

Dr. Santora will provide an overview of the NIA application process, funding opportunities, and relevant policy changes for early-career researchers.

